# Supplementary material for: Thermodynamic Correlation between Liquid–Liquid Phase Separation and Crystalline Solubility of Drug-Like Molecules
Source: Pharmaceutics. 2022 Nov 22;14(12):2560. doi: 10.3390/pharmaceutics14122560 (PMC9782016; doi:10.3390/pharmaceutics14122560)
Supplement: Supplementary file 1 [file pharmaceutics-14-02560-s001.zip › pharmaceutics-1963079-supplementary.pdf]

Thermodynamic correlation between liquid-liquid phase separation and crystalline solubility of drug-like molecules

Taiga Uekusa, Tomohiro Watanabe, Daiju Watanabe, Kiyohiko Sugano*

Molecular Pharmaceutics Lab., College of Pharmaceutical Sciences, Ritsumeikan University, 1-1-1, Noji-higashi, Kusatsu, Shiga 525-8577, Japan

* Corresponding author. Tel.: +81-77-561-2773; E-mail address: suganok@fc.ritsumei.ac.jp (K. Sugano).

1. Laser-assisted visual turbidity detection (LAVTD)

The illustration of the LAVTD device is shown in Figure S1.

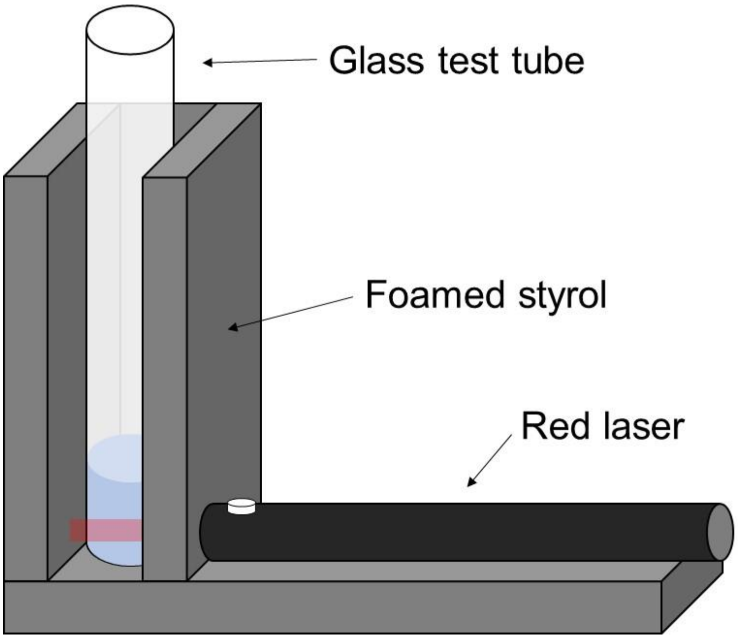


**Figure S1. Illustration of the laser-assisted visual turbidity detection device.**

2. Crystallization time of drugs.

The crystallization time for each drug is summarized in Table S1. The examples of PLM observation of crystallization time measurement are summarized in Figure S2.

**Table S1. Crystallization time of drugs at 310 K.**[1]

| Drug | < 3 sec | 3 – 10 sec | 10 – 30 sec | 30 – 60 sec |
|---|---|---|---|---|
| (S)-(+)-Naproxen | A+C | A+C | A+C | C |
| 2-Naphthoic-acid | A+C | C | C | C |
| Acemetacin | C | C | C | C |
| Benzocaine | C | C | C | C |
| Bifonazole | A | A | A | A |
| Bupivacaine | A+C | A+C | A+C | A+C |
| Carprofen | A | A | A | C |
| Chlorpromazine | A | A | A | A |
| Diclofenac [2] | A | A | C | C |
| Diphenhydramine | A | A | A | A |
| Dipyridamole | A | A | C | C |
| Fenofibrate | A | A | A | A |
| Flufenamic-acid | A | A+C | A+C | A+C |
| Flumequine | C | C | C | C |
| Flurbiprofen | A | A | C | C |
| Furosemide | A+C | A+C | C | C |
| Glipizide | A | A+C | C | C |
| Haloperidol | C | C | C | C |
| Ibuprofen [2] | A | A | A | A |
| Indomethacin | C | C | C | C |
| Ketoconazole | A | A | A | A |
| Ketoprofen | A | A | A | A |
| Ketotifen | A | A | A | A |
| Lidocaine | A | A | A | A |
| Losartan | A | A | A | A |
| Loxoprofen | A | A | A | A |
| Meclofenamic-acid | A | A | A | A |
| Mefenamic-acid | C | C | C | C |
| Meloxicam | A | A+C | C | C |
| Niflumic-acid | A+C | A+C | A+C | C |
| Orphenadrine | A | A | A | A |
| Papaverine [2] | A | A | A | A |
| Phenylbutazone | A | A | C | C |
| Phenytoin | A+C | A+C | C | C |
| Pramoxine | A | A | A | A |
| Probenecid | C | C | C | C |
| Procaine | A | A | A | A |

| | | | | |
|---|---|---|---|---|
| Propafenone [2] | A | A | A | A |
| Propranolol | A | A | A | A |
| Quinine | A | A | A | A |
| Rebamipide | A | A | A | A |
| Sulfasalazine | A | A | A | C |
| Sulindac | A | A | A | A |
| Terbinafine | A | A | A | A |
| Thioridazine | A | A | A | A |
| Verapamil | A | A | A | A |
| Warfarin [2] | A | A | C | C |

[1] A: Amorphous, C: Crystal. [2] Remained amorphous for 10 sec at 298 K.

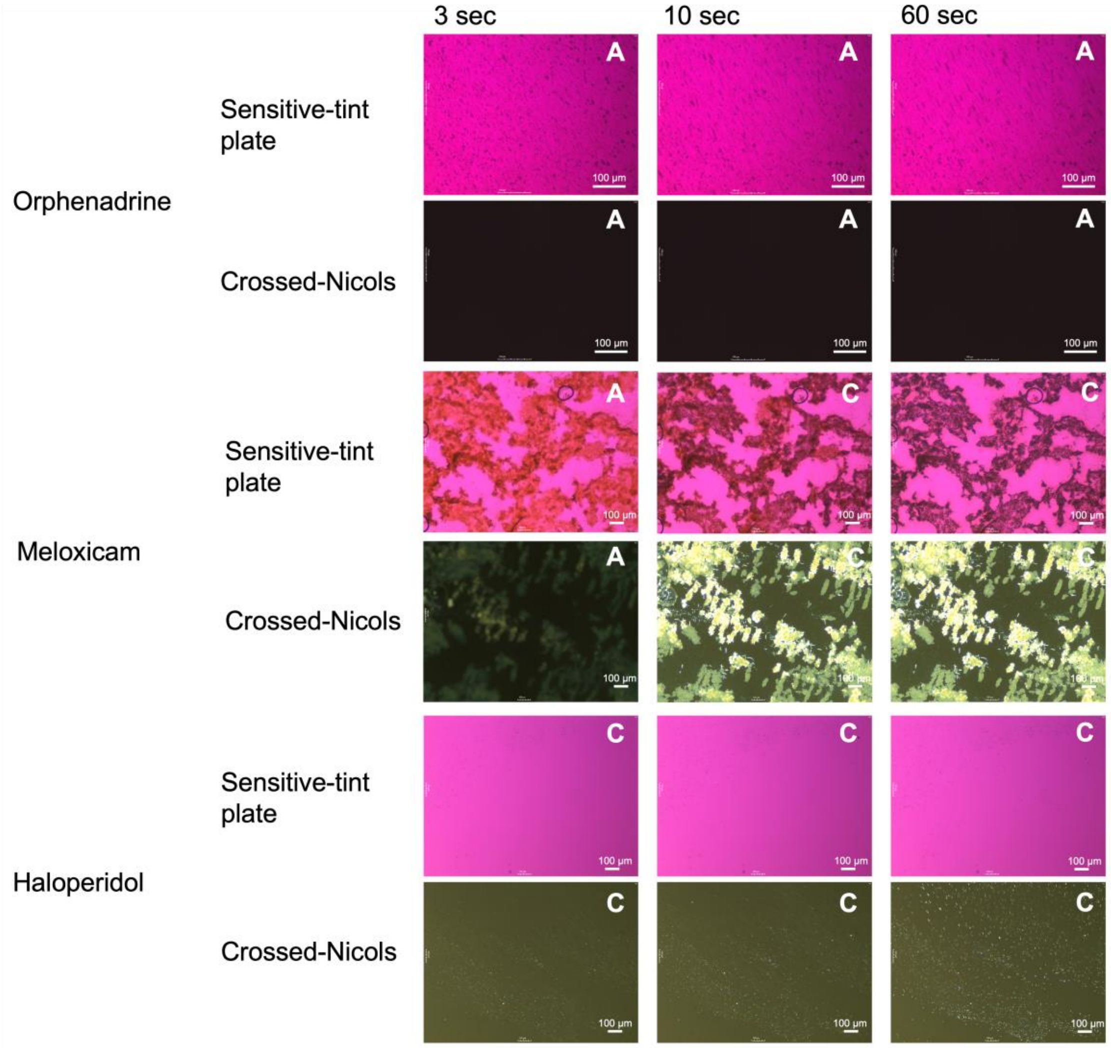


**Figure S2. The examples of PLM observation of crystallization time measurement.**

3. The chemical structure of model drugs

The chemical structure and CAS number of model drugs are summarized in Figure S3.

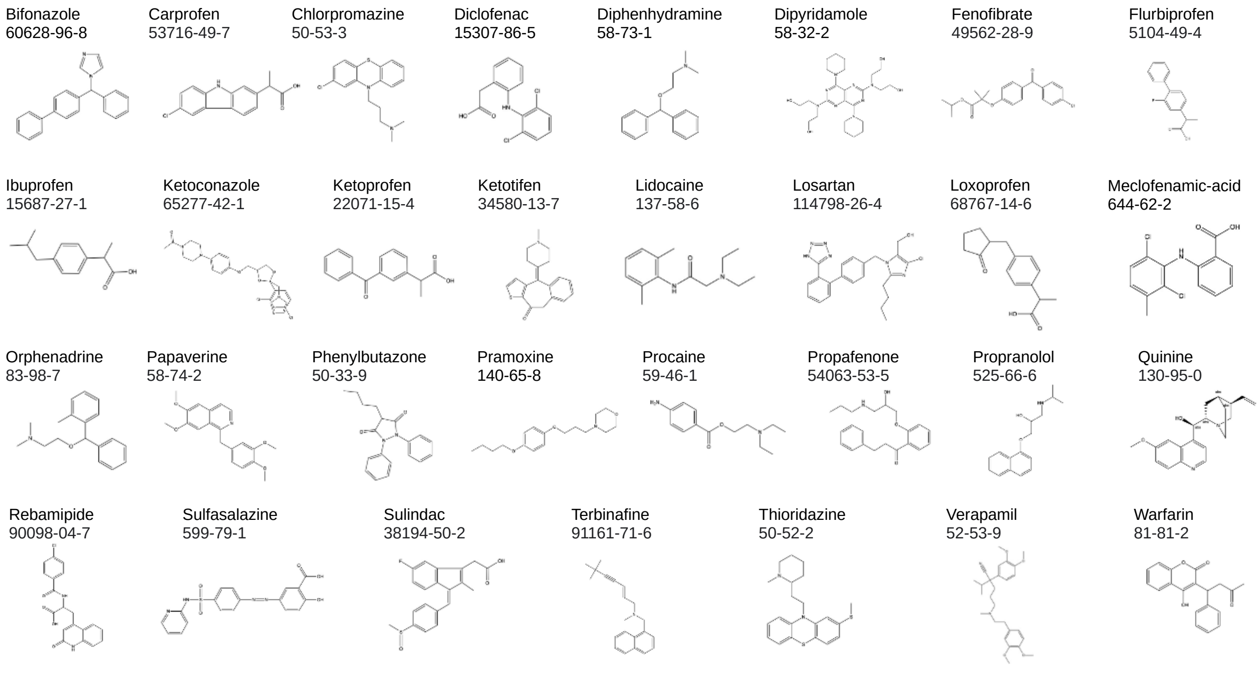


Figure S3. The chemical structure and CAS number of model drugs.

4. The intrinsic solubility ($S_0^C$) measurement.

The DSC charts of residual particles in the intrinsic solubility measurement are summarized in Figure S4. The DSC chart of the residual particles matched the initial particles except for procaine. In the case of procaine, *p*-aminobenzoic acid was produced by the decomposition of procaine and precipitated in the test tube. The pH values after reaching the equilibrium of the suspension are summarized in Table S2. The calibration curves of intrinsic solubility measurement are summarized in Figure S5.

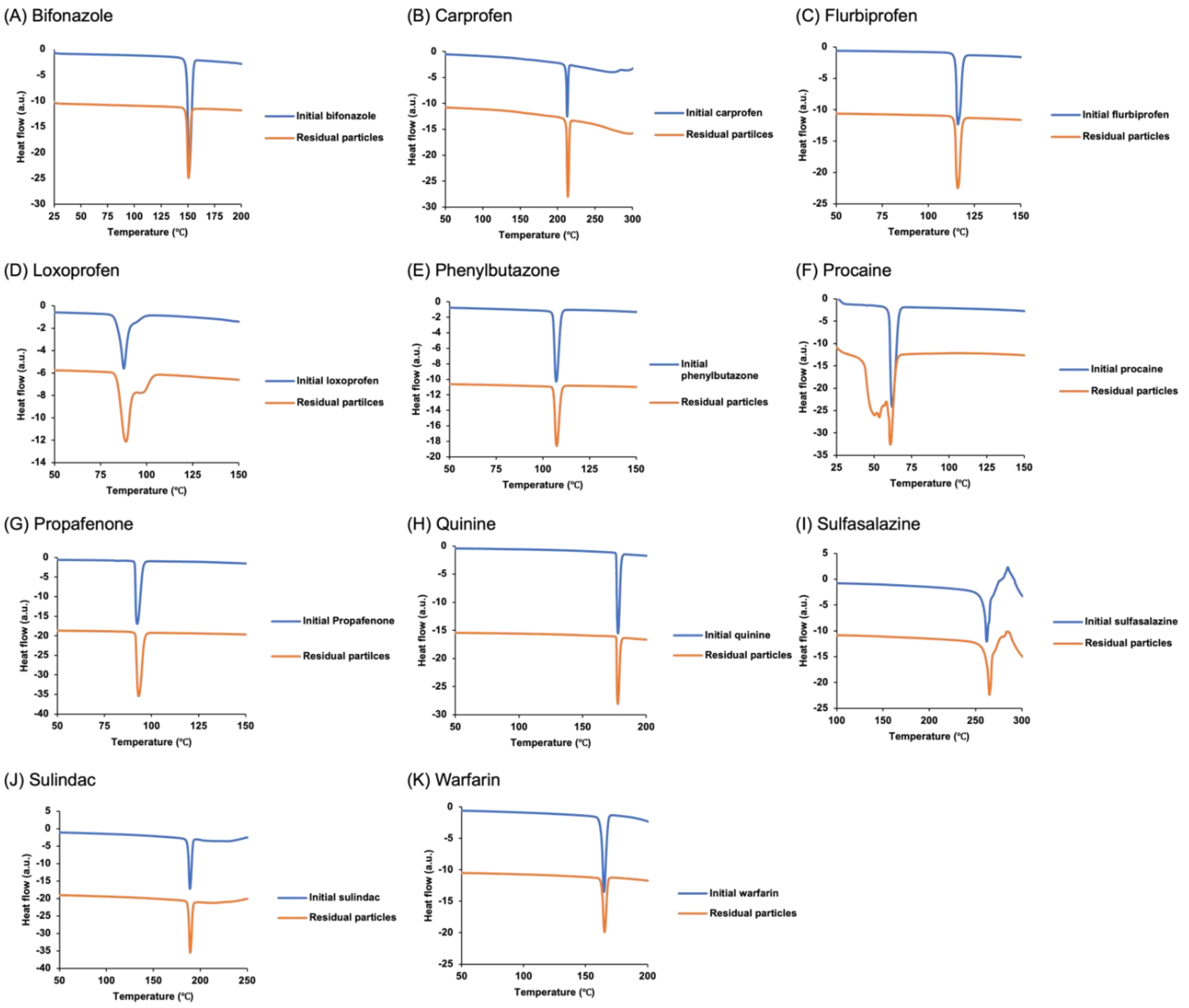


**Figure S4. DSC curves of residual particles in intrinsic solubility measurement.**

**Table S2. The pH value after reaching the equilibrium.**

| Drug | pH (mean ± S.D.) |
|---|---|
| Bifonazole | 9.0 ± 0.0 |
| Carprofen | 1.0 ± 0.0 |
| Flurbiprofen | 1.0 ± 0.0 |
| Loxoprofen | 1.0 ± 0.0 |
| Phenylbutazone | 1.1 ± 0.0 |
| Procaine | 11.6 ± 0.0 |
| Propafenone | 11.8 ± 0.0 |
| Quinine | 11.8 ± 0.0 |
| Sulfasalazine | 1.0 ± 0.0 |
| Sulindac | 1.0 ± 0.0 |

| | |
|---|---|
| Warfarin | 1.0 ± 0.0 |

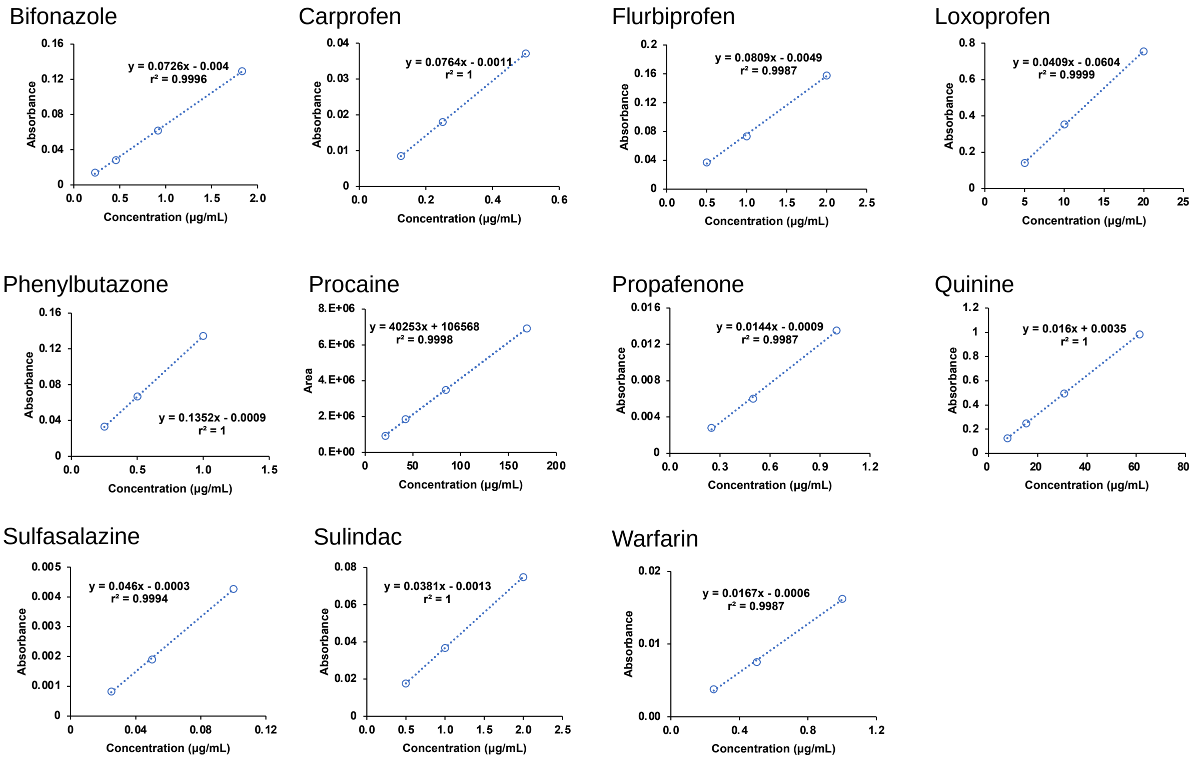


**Figure S5. Calibration curves of intrinsic solubility measurement.**
